# Xenograft-Induced Damage and Synechiae Formation in the Maxillary Sinus Mucosa: A Retrospective Histological Analysis in Rabbits

**DOI:** 10.3390/dj13100472

**Published:** 2025-10-16

**Authors:** Yasushi Nakajima, Karol Alí Apaza Alccayhuaman, Ermenegildo Federico De Rossi, Eiki Osaka, Daniele Botticelli, Erick Ricardo Silva, Samuel Porfirio Xavier, Shunsuke Baba

**Affiliations:** 1 Department of Oral Implantology, School of Dentistry, Osaka Dental University, 8-1 Kuzuhahanazonocho, Hirakata 573-1121, Osaka, Japan; y.nakajima@me.com (Y.N.); cw95zk@bma.biglobe.ne.jp (E.O.); baba-s@cc.osaka-dent.ac.jp (S.B.); 2 ARDEC Academy, 47923 Rimini, Italy; karol.ali.apazaa@gmail.com (K.A.A.A.); e.f.derossi@gmail.com (E.F.D.R.); 3 Department of Oral Biology, University Clinic of Dentistry, Medical University of Vienna, 1090 Vienna, Austria; 4 Department of Oral and Maxillofacial Surgery and Periodontology, Faculty of Dentistry of Ribeirão Preto, University of São Paulo, Ribeirão Preto 14040-904, SP, Brazil; erick.silva@usp.br (E.R.S.); spx@forp.usp.br (S.P.X.)

**Keywords:** bone regeneration, osseointegration, biomaterials, bone resorption, mucous membrane, maxillary sinus/surgery

## Abstract

**Background**: During maxillary sinus floor augmentation, the elevated sinus mucosa may come into close contact with the pristine mucosa. The presence of xenograft granules can lead to unintended mechanical and biological interactions between the two layers, and the resulting tissue damage remains poorly understood. The aim of this study was to perform a focused histological evaluation of graft-mediated interactions between the elevated and pristine sinus mucosae. **Methods**: Histological slides from five previously published rabbit sinus augmentation studies using grafts with different resorption rates were retrospectively analyzed. The following main patterns of tissue alteration were identified: (1) Proximity stage, characterized by epithelial thickening, goblet cell hyperactivity, and ciliary shortening; (2) Fusion stage, with epithelial interpenetration and loss of distinct mucosal boundaries; (3) Synechiae stage, featuring connective tissue bridges linking the two mucosae; and (4) Pristine mucosa lesions, caused by direct contact between residual graft particles and the pristine sinus mucosa. **Results**: A total of 192 sinuses were evaluated. Sinuses augmented with slowly resorbable grafts showed proximity stage in 22.3% of cases, fusion in 7.7%, direct lesions in 9.6%, and only one instance of synechia. In contrast, the faster resorbable xenograft presented only 11.1% of proximity stage, without further alterations. **Conclusions**: In this rabbit model, xenografts were associated with histological alterations of the sinus mucosa, while synechiae formation was rare. These preclinical findings should not be directly extrapolated to humans but may provide a basis for future investigations.

## 1. Introduction

Maxillary sinus floor elevation has become a widely accepted technique for augmenting bone volume in the posterior maxilla, particularly in cases where residual bone height is insufficient for implant placement [1]. Since the initial descriptions of the procedure [2,3], both lateral and transcrestal approaches have been developed and refined [4,5,6], demonstrating high clinical success rates [7,8]. Among these, the lateral approach remains the most frequently employed, offering predictable outcomes in terms of vertical bone gain [4,8].

One of the main challenges in sinus augmentation is the natural tendency of the sinus cavity to re-pneumatize, compromising the stability of the elevated space over time, as shown in both experimental [9,10] and clinical studies [11,12,13,14]. To counteract this phenomenon and preserve the augmented volume, several strategies have been proposed, including the placement of dental implants without graft material [15,16], mechanical devices [17,18,19], and various grafting materials with different resorption profiles [20,21,22,23,24].

Autogenous bone has long been considered the gold standard for sinus grafting due to its osteogenic potential, yet its high resorption rate often limits long-term volume stability [24]. In contrast, deproteinized bovine bone mineral (DBBM) has shown superior volumetric stability due to its low resorption rate and excellent osteoconductivity [25,26,27,28,29]. These features have led to their widespread application in both clinical and experimental settings [30,31,32].

Despite these advances, complications in sinus augmentation may occur [33,34] among which one of the most common remains perforation of the Schneiderian membrane [35,36,37,38,39]. Such events can compromise graft stability and negatively affect the outcome of the procedure. In some clinical reports, displacement of graft material into the maxillary sinus has been described, occasionally requiring endoscopic intervention to re-establish favorable healing conditions [40,41,42,43].

It has recently been demonstrated in experiments in rabbits that sinus membrane perforations may also occur as a delayed complication, resulting from progressive thinning of the mucosa in contact with graft particles [44]. These events appear to be strongly influenced by the type of grafting material used [45,46,47].

In particular, slowly resorbing xenografts such as deproteinized bovine bone mineral remain in close contact with the sinus mucosa for extended periods. While this persistence supports volume stability, it also increases the likelihood of mucosal thinning, perforation, or adhesion phenomena compared to faster-resorbing materials, which reduce the duration of direct contact. For this reason, slowly resorbing grafts represent a critical model for investigating graft–mucosa interactions and potential tissue alterations [44,45,46,47]. Such mucosal alterations may also predispose to long-term complications, including impaired graft stability or chronic sinus conditions. Although these sequelae remain insufficiently documented, they highlight the potential clinical importance of understanding graft-induced mucosal damage.

Although the sinus mucosa in rabbits is thinner than in humans, animal models remain essential to identify potential biological responses that may also occur clinically. Experimental studies allow controlled observation of mucosal reactions, providing insights into possible risks that warrant further confirmation in human investigations.

Moreover, recent findings have described adhesion phenomena between the elevated and pristine (non-elevated) sinus mucosae when the two surfaces come into close proximity and contact, even in the absence of graft particles [48].

**Figure 1:**
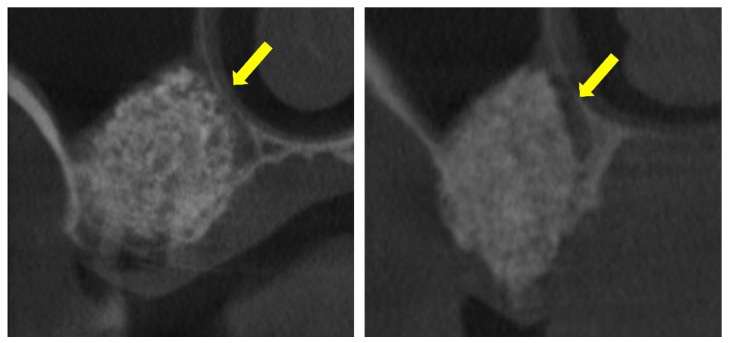
The elevated sinus mucosa is shown in direct contact with the pristine, non-elevated mucosa. The graft material appears closely positioned against the medial sinus wall, seemingly exerting pressure that maintains the two mucosal surfaces in close apposition (yellow arrows). This close apposition may contribute to the development of mucosal synechiae.

These prior studies, however, did not include a focused analysis of the interactions between the elevated and pristine sinus mucosae in cases where graft granules, positioned beneath the elevated mucosa, came into close proximity with the non-elevated mucosa (Figure 1).

## 2. Materials and Methods

### 2.1. Ethical Statement

All histological material used in this study was derived from previously approved experimental protocols carried out by the same research group at the Faculty of Dentistry of Ribeirão Preto, University of São Paulo (CEUA, Brazil). The studies were approved by the local Ethical Committee as follows:

− Amari et al., 2020 [49] (Protocol #2017.1.278.58.9, approved on 14 June 2017)
− Favero et al., 2022 [50] (Protocol #2019.1.113.58.1, approved on 8 April 2019)
− Maniwa et al., 2024 [46] (Protocol #0066/2023, approved on 14 March 2023)
− Yamada et al., 2025 [47] (Protocol #0065/2023, approved on 22 February 2023)
− Iida et al., 2016 [51] (Protocol #2015.1.834.58.7, approved on 18 November 2015)

All procedures were conducted in accordance with national regulations for animal experimentation and adhered to the ARRIVE guidelines.

### 2.2. Study Design

This investigation was designed as a retrospective histological analysis of tissue sections obtained from previously published experimental studies in rabbits. All original studies were conducted prospectively under approved ethical protocols and primarily aimed at evaluating bone formation and graft behavior after maxillary sinus augmentation. For the present work, the same histological slides were re-examined with a different focus, specifically assessing graft-mediated interactions between the elevated and pristine sinus mucosae. No new surgical procedures or animal experiments were performed for this analysis. The present study emphasizes the morphological alterations associated with graft placement and mucosal interactions, an aspect not systematically described in the original publications.

### 2.3. Sample Source

**Figure 2:**
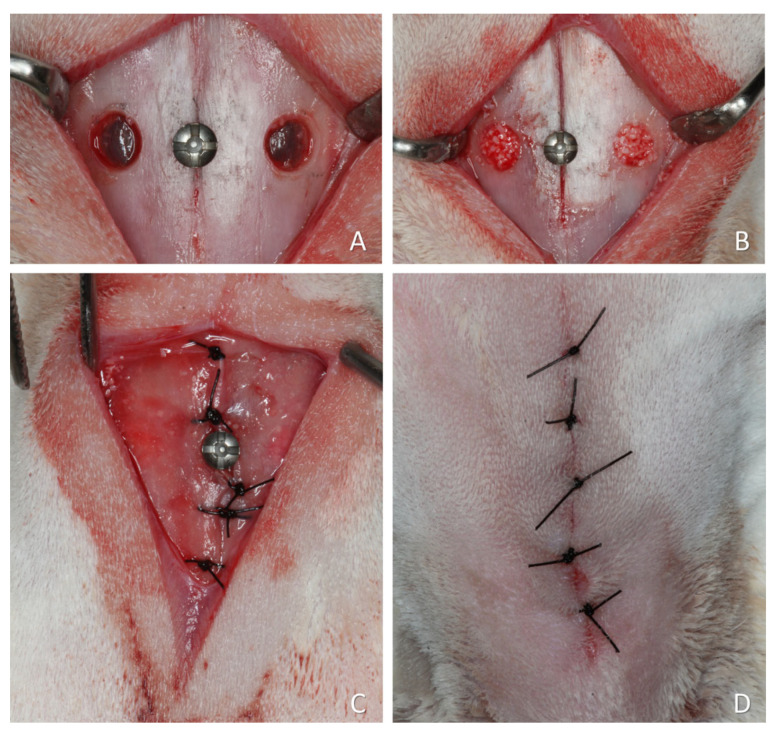
A representative surgical procedure is illustrated in Figure 2, although some variations were applied across the studies included in this article. (**A**) Two small access windows were prepared on both sides of the nasal region. A small screw was placed at the naso-incisal suture to serve as a reference point for histological processing. (**B**) The sinus mucosa was elevated, and the grafting material placed to fill the created space. (**C**) The periosteum was closed with resorbable sutures. (**D**) The wounds were closed with nylon sutures.

For the present re-analysis, four studies using slowly resorbing xenografts and one study using a faster-resorbing xenograft were included. The choice was dictated by the availability of histological slides and by the aim of evaluating whether lesions could occur in the pristine mucosa. The faster-resorbing xenograft was included only as a descriptive reference and not for statistical comparison. A representative surgical procedure is illustrated in Figure 2, although some variations were applied across the studies included in this article.

The histological slides analyzed in this retrospective study were obtained from five previously published experiments that investigated different aspects of bone regeneration and graft resorption following maxillary sinus augmentation in rabbits.

Although each study evaluated distinct biomaterials or healing periods, they shared comparable surgical models, animal management protocols, and histological processing techniques. The consistency across studies minimizes the risk of methodological bias and supports the reliability of the present retrospective analysis.

The histological material was obtained from the following published studies (for further details, please refer to the original articles):

Amari et al. (2020) [49]: This study investigated the influence of maintaining the bony window (trap-door technique) during maxillary sinus augmentation in rabbits. Eighteen animals received deproteinized bovine bone mineral (DBBM) grafts with or without the repositioning of the bony window. Healing was evaluated histologically and via microCT after 2, 4, and 8 weeks. The primary aim was to assess bone formation dynamics in relation to the presence of the window, particularly in the submucosal area. Between the first and last observation periods, the window sites showed a 25% graft loss, whereas the control sites exhibited an 18% loss.

Favero et al. (2022) [50]: This experimental study evaluated the effects of two deproteinized bovine bone grafts, one processed at low temperature (Bio-Oss^®^; Geistlich Pharma AG, Wolhusen, Switzerland) and the other at high temperature (Cerabone^®^; Botiss Biomaterials GmbH, Zossen, Germany), on sinus mucosa integrity in rabbits following sinus floor elevation. Twenty animals received bilateral sinus augmentation, with each side randomly assigned to one of the two materials. Histological evaluations were conducted at 2 and 10 weeks. Both grafts caused mucosal thinning and perforations, with no statistically significant differences between groups. Between the two healing periods, the Bio-Oss^®^ (Geistlich Pharma AG, Wolhusen, Switzerland) sites exhibited a 16% graft loss, whereas the Cerabone^®^ (Botiss Biomaterials GmbH, Zossen, Germany) sites showed a 28% loss.

Maniwa et al. (2024) [46]: This study evaluated the effect of incorporating a hyaluronic acid–polynucleotide gel (Regenfast; Geistlich Pharma AG, Wolhusen, Switzerland) into Bio-Oss^®^ (Geistlich Pharma AG, Wolhusen, Switzerland) grafts used for maxillary sinus augmentation in rabbits. Twelve animals underwent bilateral sinus lifts, and healing was assessed after 2 and 10 weeks. The study aimed to determine whether the gel enhanced bone formation or reduced sinus mucosa damage. No significant differences in new bone formation were found between test and control sites, and mucosal thinning and perforations occurred in both groups. Between the two healing periods, the Bio-Oss^®^ alone sites exhibited a 26% graft loss, whereas the Bio-Oss^®^ plus Regenfast sites showed a 37% loss.

Yamada et al. (2025) [47]: This randomized split-mouth study investigated the impact of adding 10% collagen to Bio-Oss^®^ (Geistlich Pharma AG, Wolhusen, Switzerland) on sinus mucosa integrity during sinus augmentation in rabbits. Twelve animals received the Bio-Oss^®^ (Geistlich Pharma AG, Wolhusen, Switzerland) in one sinus (control) and Bio-Oss^®^ collagen (Geistlich Pharma AG, Wolhusen, Switzerland) in the contralateral side (test). Histological analyses were performed after 2 and 12 weeks of healing. While the collagenated graft initially reduced the incidence of mucosal thinning and perforation at 2 weeks, this protective effect was no longer evident at 12 weeks, with both groups showing comparable mucosal damage. Between the two healing periods, the Bio-Oss^®^ (Geistlich Pharma AG, Wolhusen, Switzerland) sites exhibited a 31% graft loss, whereas the Bio-Oss^®^ collagen (Geistlich Pharma AG, Wolhusen, Switzerland) sites showed a 47% loss.

Given their relatively slow reduction in percentage, Bio-Oss^®^ (Geistlich Pharma AG, Wolhusen, Switzerland) and Cerabone^®^ (Botiss Biomaterials GmbH, Zossen, Germany) were classified in the present study as slowly resorbable xenografts.

Iida et al. (2019) [51]: This experimental study investigated the healing pattern after sinus mucosa elevation when a collagen membrane was placed subjacent to the sinus mucosa before grafting. Eighteen rabbits underwent bilateral sinus lifts, with a collagen membrane positioned under the sinus mucosa only at randomly assigned test sites. In both test and control sinuses, a collagenated corticocancellous porcine bone graft (Gen-Os^®^; Tecnoss^®^, Giaveno, Italy) was inserted into the elevated space, and the access window was covered with a collagen membrane. Histological analyses were performed after 2, 4, and 8 weeks of healing. The subjacent collagen membrane was not fully resorbed after 8 weeks, and no major morphometric differences were observed between groups. Only the control site was included in the analysis. Between the first and last period of observation, the control sites lost 73% of graft. Gen-Os^®^ (Tecnoss^®^, Giaveno, Italy) was therefore classified as a highly resorbable xenograft.

### 2.4. Biomaterials

Bio-Oss^®^ (Geistlich Pharma AG, Wolhusen, Switzerland) is an osteoconductive, slowly resorbing xenogeneic bone substitute derived from the mineral portion of bovine bone. It undergoes a deproteinization process at 300 °C, resulting in a highly porous, inorganic structure that closely resembles human cancellous bone in terms of physical and chemical composition [52].

Bio-Oss Collagen^®^ (Geistlich Pharma AG, Wolhusen, Switzerland) is a combination of Bio-Oss particles integrated with 10% porcine-derived type I collagen. This collagen component is resorbable and is intended to improve handling and initial graft stability during the early phases of bone regeneration.

Cerabone^®^ (Botiss Biomaterials GmbH, Zossen, Germany) consists of bovine-derived cancellous bone transformed into ceramic hydroxyapatite through thermal processing exceeding 1200 °C. It features a porous, foam-like architecture (approximately 65–80% porosity), with pore diameters ranging from 600 to 900 µm, and is characterized by high crystallinity, notable hydrophilicity, and minimal residual impurities [53,54]

Regenfast (Geistlich Pharma AG, Wolhusen, Switzerland) is a combination of hyaluronic acid and highly purified, fully resorbable polynucleotides, designed to support the regeneration of oral tissues [55].

OsteoBiol^®^ Gen-Os^®^ (Tecnoss^®^, Giaveno, Italy) is a collagenic cortico-cancellous graft obtained in the analyzed study from swine, and produced at processing temperatures not exceeding 130 °C. The biomaterial shows total and intraparticle porosities of 33.1% and 21%, respectively, with a real density of 2.43 g/cm^3^ and a mineral fraction of 64.6% [52].

### 2.5. Histological Processing

The specimens were processed at the Hard Tissue Laboratory of FORP-USP. After thorough rinsing, they were dehydrated in a graded ethanol series, with solution changes every three days under continuous agitation. The samples were then embedded in LR White™ resin and polymerized at 60 °C.

Two ground sections per sample were prepared using precision cutting and grinding equipment (Exakt^®^ system; Apparatebau, Norderstedt, Germany), initially 100–150 µm thick and subsequently reduced to 60–80 µm. The sections were stained with either Toluidine Blue or a combination of Stevenel’s Blue and Alizarin Red for histological evaluation.

### 2.6. Histological and Descriptive Analysis of Tissue Interactions

Histological evaluations were carried out under light microscopy by two experienced examiners (K.A.A.A. and D.B.). Each slide was examined multiple times and at different magnifications to ensure consistency and accuracy.

**Figure 3:**
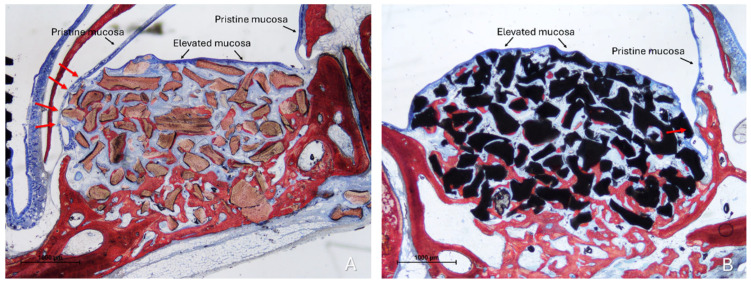
Photomicrographs of ground sections illustrating the histological outcomes of sinus floor augmentation in rabbits. (**A**) Sinus augmented with Bio-Oss^®^ (Geistlich Pharma AG, Wolhusen, Switzerland). (**B**) Sinus augmented with Cerabone^®^ (Botiss Biomaterials GmbH, Zossen, Germany). Note the graft-mediated contact between the elevated and pristine sinus mucosae, potentially resulting in damage to both layers (red arrows). Stevenel’s Blue and Alizarin Red stain.

Two distinct sinus mucosae were identified: the elevated mucosa, lining the augmented space, and the pristine mucosa, which remained attached to the native sinus walls and was not involved in the elevation procedure. Sections showing graft particles within the elevated compartment and capable of altering the structure of the elevated mucosa and inducing changes in the adjacent pristine mucosa were selected for qualitative description (Figure 3).

The main patterns of tissue alteration were identified in all histological slides, according to a previously described categorization [48]: (1) Proximity stage, characterized by epithelial thickening, goblet cell hyperactivity, and ciliary shortening; (2) Fusion stage, defined by epithelial interpenetration and the loss of distinct mucosal boundaries; and (3) Synechiae stage, featuring connective tissue bridges connecting the two mucosae. In addition, an additional alteration was observed: (4) Lesions to the pristine mucosa, caused by direct contact of the residual graft with the elevated mucosa.

## 3. Results

### Histological Findings

**Figure 4:**
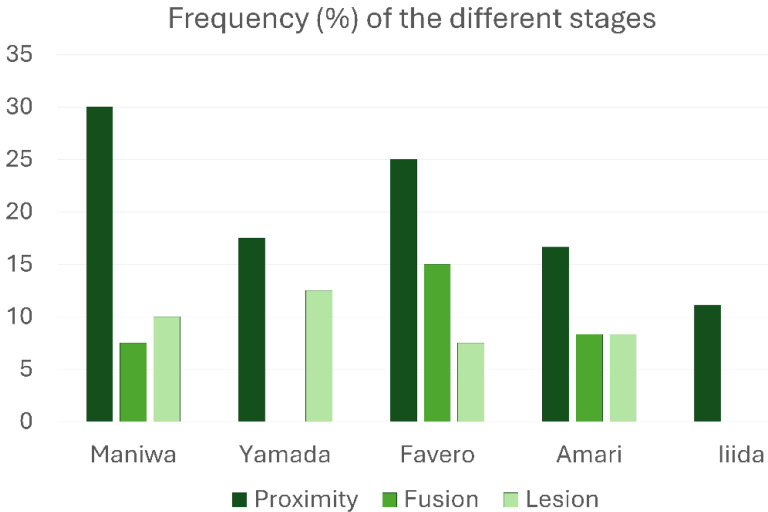
Graphs illustrating the percentage frequency of occurrence of the various stages of interaction between the elevated and pristine sinus mucosae.

All types of interactions between the elevated and pristine sinus mucosae mediated by graft particles, as indicated in the categorization, were observed. Specifically, proximity stages were identified in at least one site of 37 out of 174 sinuses (21.3%), fusion stages in 12 sinuses (6.9%), and lesions in 15 sinuses (8.6%). Only one case of synechia was recorded. The most frequent stage was proximity, whereas a remarkable percentage of lesions was detected in all groups treated with low-resorption grafts (Table 1; Figure 4). Interestingly, in the high-resorption graft group [51], only proximity stages were observed, with no other types of lesions to the pristine mucosa.

**Figure 5:**
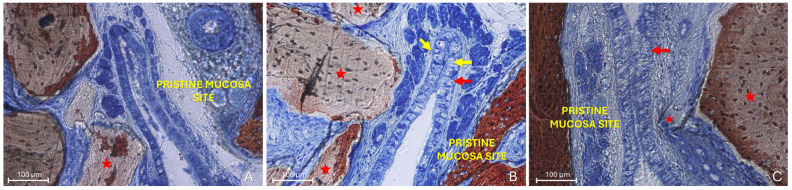
Photomicrographs of ground sections illustrating examples of the “Proximity stage”. (**A**) A Bio-Oss^®^ granule (Geistlich Pharma AG, Wolhusen, Switzerland; red star) displaces mucous glands and maintains the elevated sinus mucosa in close vicinity to the pristine mucosa, with interposed mucus. (**B**) Elevated and pristine mucosae in close contact within a folding region; note the entangled cilia, the hypertrophic activity of the pristine mucosa epithelium (red arrows) and the increased number and activity of goblet cells (yellow arrows). (**C**) Proximity stage observed outside the folding region, showing cilia from both mucosae entangled within mucus. Note a small granule embedded within the lamina propria of the elevated mucosa (small red star) and the hypertrophic/hyperplastic activity of the elevated mucosa (red arrow). Stevenel’s Blue and Alizarin Red stain.

The “Proximity stage” was characterized by close apposition or contact between the two mucosal layers, often with interposed mucus (Figure 5A).

The pseudostratified ciliated columnar epithelium generally showed no significant alterations, except for an increase in the average height of columnar cells, likely associated with enhanced mitotic activity (Figure 5B). In some regions, an increased number and activity of goblet cells were also identified (Figure 5B). Within these contact areas, the cilia of both epithelial surfaces appeared shortened and entangled within the surrounding mucus (Figure 5B,C). Graft granules appeared to maintain in close contact the two mucosae, while displacing vessels and mucous glands.

**Figure 6:**
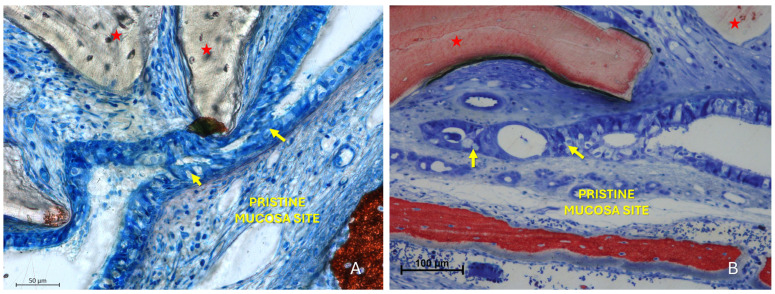
Photomicrographs of ground sections illustrating fusion stages. (**A**) Advanced stages of mucosal interaction characterized by interpenetration of epithelial cells from both mucosal layers, making the boundary between them indistinct (yellow arrows). A Bio-Oss^®^ granule (Geistlich Pharma AG, Wolhusen, Switzerland; red star) displaces the elevated mucosa against the pristine one. (**B**) Fusion stage with a Bio-Oss^®^ particle (Geistlich Pharma AG, Wolhusen, Switzerland; red star) in close proximity to the elevated mucosa. The contact between the elevated and pristine mucosa led to fusion of the two epithelial layers (yellow arrow), rendering the distinction between them impossible. Stevenel’s Blue and Alizarin Red stain.

**Figure 7:**
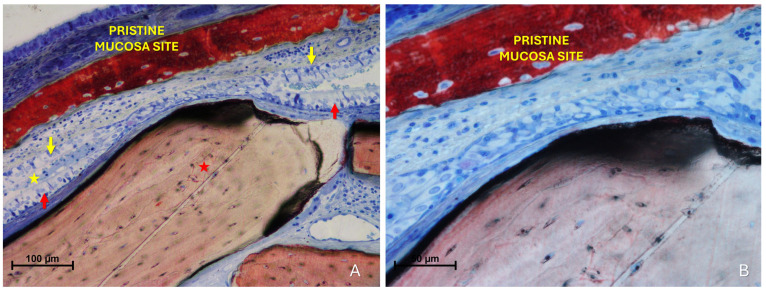
Photomicrographs of ground sections illustrating fusion stages. (**A**) The epithelial layers appear not simply in contact but structurally integrated, making the boundaries between the opposing epithelia indistinguishable. Note the hypertrophy of the pseudostratified ciliated columnar epithelium in both the pristine (yellow arrows) and the elevated mucosa (red arrows). Mucus is entrapped between the two epithelial layers (yellow stars). (**B**) Higher magnification of the area of interest. Stevenel’s Blue and Alizarin Red stain.

**Figure 8:**
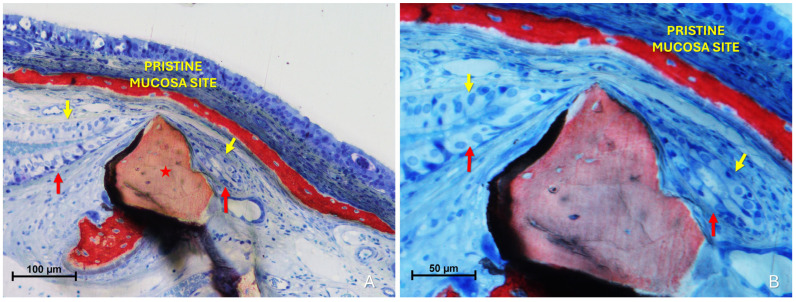
Photomicrographs of ground sections illustrating fusion stages. (**A**) A Bio-Oss^®^ granule (Geistlich Pharma AG, Wolhusen, Switzerland; red star) deviates the elevated mucosa (red arrows) against the pristine mucosa (yellow arrows). (**B**) The folding region is sequestered to the right of the Bio-Oss^®^ granules (Geistlich Pharma AG, Wolhusen, Switzerland).

The “Fusion stage” represented a subsequent phase in which the interacting areas showed interpenetration of epithelial cells from both mucosal surfaces (Figure 6, Figure 7 and Figure 8). The epithelial layers were not merely in contact but appeared structurally integrated, making it difficult to distinguish the boundaries between the opposing epithelia. Graft particles seemed to compress the elevated mucosa against the pristine one, possibly facilitating this fusion. In addition, bridges of epithelial cells connecting the two mucosae were observed, delimiting enclosed regions within the sinus cavity, potentially with a tubular configuration.

**Figure 9:**
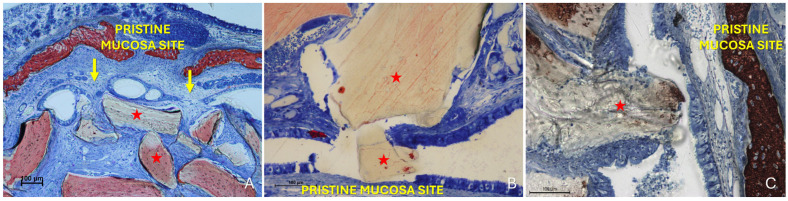
(**A**) Photomicrograph of a ground section illustrating a synechia stage, where bridges of connective tissue divide and isolate sinus regions (yellow arrows); red stars indicate some of the granules. (**B**) Direct damage to the pristine mucosa caused by Bio-Oss^®^ granules (Geistlich Pharma AG, Wolhusen, Switzerland; red star) penetrating through the perforated elevated mucosa. (**C**) A Bio-Oss^®^ granule (Geistlich Pharma AG, Wolhusen, Switzerland; red star) that penetrated the elevated mucosa and disrupted the adjacent pristine mucosa. Note the epithelium adhering to the granule in an attempt to preserve mucosal integrity.

The “Synechiae stage” was characterized by the formation of connective tissue bridges lacking epithelial lining, which connected the elevated mucosa with adjacent regions of the sinus wall. Graft particles were often embedded within these fibrous structures (Figure 9A).

More severe damage to the pristine (non-elevated) mucosa was observed in areas where the elevated sinus mucosa had been perforated by graft granules, again without direct inter-mucosal contact. In these cases, the injury could be limited to the epithelial layer or involving the underlying lamina propria (Figure 9B,C).

**Figure 10:**
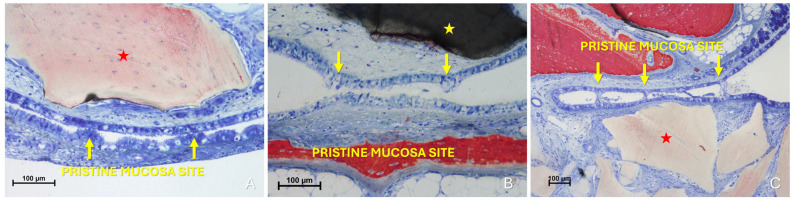
Photomicrographs of ground sections showing hypermitotic growths, originating from one or both mucosal surfaces, that developed as protruding excrescences in proximity to xenograft granules (Bio-Oss^®^, Geistlich Pharma AG, Wolhusen, Switzerland, red star; Cerabone^®^, Botiss Biomaterials GmbH, Zossen, Germany, yellow star). (**A**) Hypertrophic/hyperplastic formations extending from the pristine mucosa toward the elevated mucosa. (**B**) Hypertrophic/hyperplastic formations from both mucosae appear to be mutually attracted. (**C**) Epithelial bridges formed, connecting the two mucosae. Stevenel’s Blue and Alizarin Red stain.

Histological analysis clearly revealed long-distance interactions between the elevated and pristine mucosa (Figure 10A–C). Hypertrophic/hyperplastic activity, originating from one or both mucosal surfaces, developed as protruding excrescences. In cases where both surfaces exhibited such growths, they appeared to approach each other as if mutually attracted, eventually forming connective bridges. The biomaterial granules contributed to maintaining the two mucosae in close proximity, thereby facilitating these interactions.

## 4. Discussion

This study aimed to investigate a previously underreported aspect of sinus augmentation procedures, focusing on the histological evaluation of graft-mediated interactions between the elevated and pristine sinus mucosae. The findings revealed a range of tissue responses, from adhesion phenomena between the two mucosal layers to instances of damage affecting both the elevated and the pristine mucosae.

The close contact between the graft granules and the elevated sinus mucosa may create conditions conducive to a dislocation of vessels and glands and a progressive thinning of the mucosal layer, potentially leading to its perforation. This may result in the displacement of graft particles from the augmented space and a subsequent attempt by the sinus mucosa to restore the integrity of the inner cavity by closing the affected area, as shown in other reports [44,45,46,47].

Studies have demonstrated that the occurrence of mucosal thinning and perforation is strongly influenced by the characteristics of the graft material, in particular by its resorption rate. In a rabbit model, a collagenic xenograft with a higher resorption rate led to fewer thinning sites and almost no perforations compared to deproteinized bovine bone matrix [45].

In a further study in rabbits, the mucosal response to two xenografts processed at different temperatures (approximately 300–1200 °C) was compared [50]. The frequency of mucosal thinning and perforations did not differ significantly between groups, supporting the hypothesis that the material’s resorption dynamics, rather than its origin or processing temperature alone, may play a more critical role in determining mucosal damage.

Adhesion phenomena between the elevated and pristine sinus mucosae were first described by Nakajima et al. in a rabbit model [48]. In that study, three distinct stages of adhesion were identified: the Proximity stage, characterized by close apposition of the mucosae with minor epithelial changes and cilia entangled in mucus; the Fusion stage, marked by interpenetration and structural integration of epithelial layers; and the Synechiae stage, defined by the formation of connective tissue bridges devoid of epithelial lining.

In the present study, we investigated the interaction between graft granules and adhesion phenomena and the influence on the pristine mucosa. All three stages were observed, with the granules appearing to play a key role in maintaining contact between the elevated and pristine mucosae, thereby facilitating adhesion progression. Several Proximity stages were detected in all studies, mainly located near the folding region. Fusion stages were also identified in three out of four studies using grafts with a low resorptive rate, while all four studies revealed a high number of direct damages to the pristine mucosa caused by the granules. Within this group of slowly resorbing xenografts no qualitative differences in the type or severity of mucosal alterations were observed, as Proximity, Fusion, and Lesion stages occurred with similar frequency across these materials. Conversely, the xenograft with a high resorptive rate did not exhibit any of the more advanced interactions between the two mucosae, such as Fusion or direct Lesion. These findings confirm the higher risk of mucosal damage when using slowly resorbable compared to highly resorbable filler materials.

The present study evaluated articles that used xenografts with either high or low resorption rates, as substantiated by the data reported in the respective studies, and these results are consistent with findings from systematic reviews [56,57].

A previous investigation demonstrated that highly resorbable xenografts are associated with reduced elevated mucosal damage compared to DBBM [45]. In this context, the addition of a subjacent collagen membrane did not provide any protective advantage. On the contrary, our analysis on the damage to the pristine mucosa revealed two proximity sites in the control group and eight in the test group with the membrane, possibly due to the increased volumetric expansion of the membrane during the early phases of healing. However, these sites with the subjacent membrane were not included in the present analysis, since only the control sites were considered to ensure comparability with the other studies.

Therefore, within the limits of the available data, the use of collagen membranes placed beneath the sinus mucosa cannot be recommended as a protective measure when highly resorbable grafts are employed. At present, no evidence supports their necessity when slowly resorbable grafts are used.

A noteworthy finding was the indirect interaction observed between the elevated mucosa and the opposing pristine mucosa, which, despite the absence of direct contact, still showed signs of being affected. The elevated and pristine mucosae, although merely in close proximity, appeared to exhibit a coordinated epithelial response, characterized by epithelial hypertrophy/hyperplasia, possibly reflecting an attempt to bridge the gap and reestablish continuity. This finding confirmed similar observations reported by Nakajima et al. [48].

In some cases (Figure 9C), the sinus mucosa attempted to maintain the integrity of the elevated space, with the epithelium adhering to graft particles, seemingly as a means to preserve the compartment. Similar reparative tendencies have been reported in several studies [44,45,46,47] and were particularly emphasized in an investigation where large intentional perforations in the sinus mucosa showed substantial epithelial repair after two weeks when filled with a collagen-based xenograft [58]. Another study investigated the healing process following repair of sinus mucosa perforations using a collagen membrane [59]. Although in the present study we did not observe clear outgrowth of new mucosal tissue enveloping the graft, the adhesion of the epithelium around granules indicates an effort to stabilize the compartment internally. These observations should not be seen as contradictory but rather as different manifestations—damage and repair—within the dynamic biological response of the Schneiderian membrane to graft contact.

Our histological findings clearly showed that xenograft granules can perforate the elevated sinus mucosa and cause direct damage to the pristine mucosa, representing a tangible biological risk in this model. Fusion between mucosal layers or the formation of synechiae could further alter sinus physiology, although such consequences remain speculative in the absence of functional data.

Another important observation was that damage to the pristine mucosa could occur even in the absence of direct graft contact. These subtle interactions highlight the complex biomechanical and cellular responses of the Schneiderian membrane to sinus augmentation. These findings expand our understanding of the sinus mucosa’s behavior in response to biomaterials and mechanical stimuli following elevation procedures. The observed stages, ranging from non-invasive epithelial adaptations to complete connective tissue bridges and tissues damages suggest a spectrum of tissue reactions that may occur depending on the degree of contact, pressure, and tissue integrity.

The present findings highlight a potential risk associated with slowly resorbable grafting materials, which in rabbits induced thinning and, in some cases, perforation of the sinus mucosa. Although these outcomes cannot be directly extrapolated to humans due to anatomical, physiological, and biomechanical differences, the progressive nature of the damage observed suggests that similar adverse effects cannot be excluded. Animal research thus plays a crucial role in uncovering biological processes that may remain unrecognized, providing early evidence of possible complications and generating hypotheses that warrant confirmation in human studies.

Nevertheless, as with all preclinical studies, a key limitation of the present investigation is the use of a rabbit model. It may be argued that the sinus mucosa in rabbits is thinner (~100–200 µm) [47] compared to that of humans. However, a clinical study reported that more than 30% of patients presented a mucosal thickness of <1 mm [60]. Moreover, thinning and perforation should be regarded as progressive phenomena, as demonstrated in several experimental studies [44,45,46,47]. This progressive pattern makes it unlikely that the observed alterations were caused by surgical trauma alone. Indeed, a single intraoperative perforation would not explain subsequent histological changes such as displacement of vessels and mucous glands, reduction in mucosal thickness, epithelial disruption, or cilia loss, which have been consistently reported [44,45,46,47]. In addition, although particle size and morphology were not the primary focus of the present analysis, these features may also play a role in mucosal damage, as suggested by previous reports, and should be considered in future studies.

The clinical significance of these events is therefore uncertain and likely depends on the extent and localization of the lesions. In support of this interpretation, CBCT images from clinical cases were included, showing instances where the elevated sinus mucosa appeared adherent to the pristine mucosa, apparently sustained by interposed graft material. These observations do not demonstrate functional impairment, but they suggest that the histological alterations observed in rabbits may also occur in humans and therefore deserve further investigation. While limited adhesions may favor compartmental healing, extensive involvement could hypothetically disrupt sinus physiology and compromise graft preservation, hypotheses that warrant confirmation in future studies.

Another limitation of this study is its retrospective nature and reliance on histological material from experiments originally designed for different primary objectives. Nevertheless, the consistency of surgical techniques and histological processing supports the reliability of the observations.

Another limitation of the present study is that it was not designed as a comparative investigation. Although one set of slides from a faster resorbable xenograft was included as a descriptive reference, this cannot be considered a true control group. In principle, a graft-free group would allow a clearer separation between surgical trauma and graft-related effects. However, it has been shown that graft-free sinus elevation does not maintain the augmented compartment, since the mucosa tends to return to its original position shortly after surgery [9]. For this reason, the use of a highly resorbable xenograft may represent a more informative descriptive reference than an ungrafted sinus, although it still cannot replace a true control. A proper comparative evaluation of different biomaterials would require a specifically designed study with balanced groups and predefined outcomes.

The absence of a graft-free control group represents another limitation of the present study. Indeed, several clinical investigations have demonstrated favorable outcomes of implants placed simultaneously with sinus floor elevation without the use of grafting materials [61,62,63,64,65,66]. However, in a rabbit study where sinus floor augmentation with simultaneous implant placement was performed, 24 out of 36 sinuses showed large perforations of the sinus mucosa at the implant apex, with additional lesions along the implant threads [67]. Therefore, this study could not be considered a reliable control and was not included in the analysis.

Finally, although the included studies provided well-defined healing periods, the retrospective design did not allow for balanced statistical comparisons across different materials and time points. Future prospective studies specifically designed for this purpose will be necessary to establish such correlations.

Further research is warranted to clarify whether the tissue-level phenomena observed in this study have clinical significance. On the experimental side, advanced three-dimensional imaging techniques (e.g., micro-CT, confocal microscopy) and in vivo functional assessments (such as evaluations of mucociliary transport or pressure dynamics) may help elucidate the mechanisms and progression of mucosal alterations. On the clinical side, prospective studies and long-term radiographic or endoscopic follow-ups could be valuable in detecting sinus membrane changes, graft displacement, or impaired sinus function in patients undergoing maxillary sinus augmentation. In particular, it remains to be clarified whether the localized ciliary loss observed in preclinical models has any measurable impact on overall sinus physiology, or whether compensatory mechanisms in the remaining mucosa render these alterations clinically irrelevant.

Finally, we acknowledge that human studies directly addressing these tissue-level phenomena are lacking. The present investigation therefore represents an initial step, and future clinical research will be essential to verify whether similar progressive alterations may occur in patients undergoing sinus augmentation.

## 5. Conclusions

Within the limitations of this rabbit model, xenografts were associated with histological alterations of the sinus mucosa, including epithelial disruption, mucosal fusion between elevated and pristine layers, and direct lesions to the pristine mucosa. Synechiae formation appeared to be a rare event. These preclinical findings highlight possible biological risks that warrant further investigation in specifically designed studies.

## Figures and Tables

**Table 1:** Frequency (%) of the different stages of interaction between the elevated and pristine sinus mucosae. A single case of synechiae was observed in the study by Amari et al.

Study	Resorption Rate	Proximity	Fusion	Lesion
Maniwa et al. [46]	Low	30	7.5	10
Yamada et al. [47]	Low	17.5	0	12.5
Favero et al. [50]	Low	25	15	7.5
Amari et al. [49]	Low	16.7	8.3	8.3
Iida et al. [51] (only control)	High	11.1	0	0

## Data Availability

The data supporting the findings of this study are available from the corresponding author upon reasonable request.

## References

[1] Chen J., Lu Y., Xu J., Hua Z. (2024). Clinical evaluation of maxillary sinus floor elevation with or without bone grafts: A systematic review and meta-analysis of randomised controlled trials with trial sequential analysis. Arch. Med. Sci..

[2] Boyne P.J., James R.A. (1980). Grafting of the maxillary sinus floor with autogenous marrow and bone. J. Oral Surg..

[3] Tatum H. (1986). Maxillary and sinus implant reconstructions. Dent. Clin. N. Am..

[4] Lundgren S., Cricchio G., Hallman M., Jungner M., Rasmusson L., Sennerby L. (2016). Sinus floor elevation procedures to enable implant placement and integration: Techniques, biological aspects and clinical outcomes. Periodontology 2000.

[5] Summers R.B. (1994). A new concept in maxillary implant surgery: The osteotome technique. Compendium.

[6] Pjetursson B.E., Lang N.P. (2014). Sinus floor elevation utilizing the transalveolar approach. Periodontology 2000.

[7] Del Fabbro M., Wallace S., Testori T. (2013). Long-Term Implant Survival in the Grafted Maxillary Sinus: A Systematic Review. Int. J. Periodontics Restor. Dent..

[8] Pjetursson B.E., Tan W.C., Zwahlen M., Lang N.P. (2008). A systematic review of the success of sinus floor elevation and survival of implants inserted in combination with sinus floor elevation. J. Clin. Periodontol..

[9] Asai S., Shimizu Y., Ooya K. (2002). Maxillary sinus augmentation model in rabbits: Effect of occluded nasal ostium on new bone formation. Clin. Oral Implant. Res..

[10] Xu H., Shimizu Y., Asai S., Ooya K. (2004). Grafting of deproteinized bone particles inhibits bone resorption after maxillary sinus floor elevation. Clin. Oral Implant. Res..

[11] Xiao P., Chen C., Shen X., Xu A., Sharaf M.A., Lu H., He F. (2024). Bone volume and height changes for lateral window sinus floor elevation using two types of deproteinized bovine bone mineral: A retrospective cohort study of 1–4 years. Clin. Oral Implant. Res..

[12] Zhang L., Si M., Shi J., Yang G., Shi Y. (2019). Evaluation of three-dimensional contraction of the volume of grafts after staged augmentation of the sinus floor, and an analysis of influential factors. Br. J. Oral Maxillofac. Surg..

[13] Lewin S., Riben C., Thor A., Öhman-Mägi C. (2019). Bone Volume Assessment Around Dental Implants After Open Maxillary Sinus Elevation Surgery: A Quantitative Approach to CBCT Images. Int. J. Oral Maxillofac. Implant..

[14] Temmerman A., Van Dessel J., Cortellini S., Jacobs R., Teughels W., Quirynen M. (2017). Volumetric changes of grafted volumes and the Schneiderian membrane after transcrestal and lateral sinus floor elevation procedures: A clinical, pilot study. J. Clin. Periodontol..

[15] Fettouh A.I.A., Ghallab N.A., Adel N., Nasser R., Gamal N., Samy M., Shemais N. (2025). Graftless Sinus Floor Elevation Using the Lateral or Transcrestal Approach. A Randomized Clinical Trial with One Year Follow-Up. Clin. Oral Implant. Res..

[16] Ye M., Liu W., Chen Z., Yan L., Lin X., Wang H.L. (2025). Lateral Sinus Floor Elevation Without Bone Graft: A Single- Center Retrospective Study of 216 Implants with a Mean 4-Year Follow-Up. Int. J. Oral. Maxillofac. Implant..

[17] Cricchio G., Palma V.C., Faria P.E., De Oliveira J.A., Lundgren S., Sennerby L., Salata L.A. (2009). Histological Findings Following the Use of a Space-Making Device for Bone Reformation and Implant Integration in the Maxillary Sinus of Primates. Clin. Implant. Dent. Relat. Res..

[18] Cricchio G., Palma V.C., Faria P.E., de Olivera J.A., Lundgren S., Sennerby L., Salata L.A. (2009). Histological Outcomes on the Development of New Space-making Devices for Maxillary Sinus Floor Augmentation. Clin. Implant. Dent. Relat. Res..

[19] Johansson L., Isaksson S., Adolfsson E., Lindh C., Sennerby L. (2012). Bone Regeneration Using a Hollow Hydroxyapatite Space--Maintaining Device for Maxillary Sinus Floor Augmentation—A Clinical Pilot Study. Clin. Implant. Dent. Relat. Res..

[20] Zhang Y., Yang S.-S., Zhang N.-N., Huang G.-L. (2025). Effect of platelet-derived bone enhancers used as adjuncts to deproteinized bovine bone matrix in maxillary sinus floor elevation: A systematic review and meta-analysis. BMC Oral Health.

[21] Alkandari M., Alkandari M., Alhallaq R., Mohammad M., Safar D., Alobaidan A., Alobaidan M., Albusarah S., Enki A., Malallah J. (2025). Biphasic Calcium Phosphate Versus Deproteinized Bovine Bone Mineral for Sinus Floor Elevation: A Systematic Review and Meta-Analysis of Randomized Controlled Trials. Cureus.

[22] Ghodsian D., D’jesús S., Sánchez-Labrador L., Cobo-Vázquez C.M., Brinkmann J.C.-B., Martínez-González J.M., Meniz-García C. (2024). Maxillary Sinus Augmentation with Autogenous Tooth Grafting Material: A Systematic Review. Biomimetics.

[23] Guo T., Gu Y., Zhang X., Ding X., Zhang X., Zhu Y., Mo J., Shi J., Lai H. (2024). Bovine-originated xenografts versus synthetic bone grafting materials in lateral maxillary sinus floor augmentation: A systematic review and meta-analysis. Clin. Implant. Dent. Relat. Res..

[24] Trimmel B., Gede N., Hegyi P., Szakács Z., Mezey G.A., Varga E., Kivovics M., Hanák L., Rumbus Z., Szabó G. (2020). Relative performance of various biomaterials used for maxillary sinus augmentation: A Bayesian network meta-analysis. Clin. Oral Implant. Res..

[25] Jensen T., Schou S., Svendsen P.A., Forman J.L., Gundersen H.J.G., Terheyden H., Holmstrup P. (2011). Volumetric changes of the graft after maxillary sinus floor augmentation with Bio-Oss and autogenous bone in different ratios: A radiographic study in minipigs. Clin. Oral Implant. Res..

[26] Manfro R., Fonseca F.S., Bortoluzzi M.C., Sendyk W.R. (2013). Comparative, Histological and Histomorphometric Analysis of Three Anorganic Bovine Xenogenous Bone Substitutes: Bio-Oss, Bone-Fill and Gen-Ox Anorganic. J. Maxillofac. Oral Surg..

[27] Moon J.-W., Sohn D.-S., Heo J.-U., Kim J.S. (2015). Comparison of Two Kinds of Bovine Bone in Maxillary Sinus Augmentation. Implant. Dent..

[28] Mordenfeld A., Lindgren C., Hallman M. (2015). Sinus Floor Augmentation Using Straumann^®^ BoneCeramic™ and Bio-Oss^®^ in a Split Mouth Design and Later Placement of Implants: A 5-Year Report from a Longitudinal Study. Clin. Implant. Dent. Relat. Res..

[29] Sbordone L., Levin L., Guidetti F., Sbordone C., Glikman A., Schwartz-Arad D. (2010). Apical and marginal bone alterations around implants in maxillary sinus augmentation grafted with autogenous bone or bovine bone material and simultaneous or delayed dental implant positioning. Clin. Oral Implant. Res..

[30] Stumbras A., Krukis M.M., Januzis G., Juodzbalys G. (2019). Regenerative bone potential after sinus floor elevation using various bone graft materials: A systematic review. Quintessence Int..

[31] Galindo-Moreno P., de Buitrago J.G., Padial-Molina M., Fernández-Barbero J.E., Ata-Ali J., O Valle F. (2018). Histopathological comparison of healing after maxillary sinus augmentation using xenograft mixed with autogenous bone versus allograft mixed with autogenous bone. Clin. Oral Implant. Res..

[32] Busenlechner D., Huber C.D., Vasak C., Dobsak A., Gruber R., Watzek G. (2009). Sinus augmentation analysis revised: The gradient of graft consolidation. Clin. Oral Implant. Res..

[33] Lyu M., Xu D., Zhang X., Yuan Q. (2023). Maxillary sinus floor augmentation: A review of current evidence on anatomical factors and a decision tree. Int. J. Oral Sci..

[34] Park W.-B., Okany K.P., Park W., Han J.-Y., Lim H.-C., Kang P. (2024). Atypical and Late-Developed Sinus Graft Complications Following Maxillary Sinus Augmentation: Successful Management with Guided Bone Regeneration. Medicina.

[35] Kim J., Jang H. (2019). A review of complications of maxillary sinus augmentation and available treatment methods. J. Korean. Assoc. Oral. Maxillofac. Surg..

[36] Hsu Y., Rosen P.S., Choksi K., Shih M., Ninneman S., Lee C. (2022). Complications of sinus floor elevation procedure and management strategies: A systematic review. Clin. Implant. Dent. Relat. Res..

[37] Masri D., Jonas E., Ghanaiem O., Chaushu L. (2025). Schneiderian membrane perforation repair using a crosslinked collagen membrane: A retrospective cohort study. Head Face Med..

[38] Stacchi C., Andolsek F., Berton F., Perinetti G., Navarra C., Di Lenarda R. (2017). Intraoperative Complications During Sinus Floor Elevation with Lateral Approach: A Systematic Review. Int. J. Oral Maxillofac. Implant..

[39] Atieh M., Alsabeeha N., Tawse-Smith A., Faggion C., Duncan W. (2015). Piezoelectric Surgery vs. Rotary Instruments for Lateral Maxillary Sinus Floor Elevation: A Systematic Review and Meta-Analysis of Intra- and Postoperative Complications. Int. J. Oral Maxillofac. Implant..

[40] A Urban I., Nagursky H., Church C., Lozada J.L. (2012). Incidence, diagnosis, and treatment of sinus graft infection after sinus floor elevation: A clinical study. In. J. Oral. Maxillofac. Implant..

[41] Park W., Han J., Kang P., Momen-Heravi F. (2019). The clinical and radiographic outcomes of Schneiderian membrane perforation without repair in sinus elevation surgery. Clin. Implant. Dent. Relat. Res..

[42] Galli S.K.D., Lebowitz R.A., Giacchi R.J., Glickman R., Jacobs J.B. (2001). Chronic Sinusitis Complicating Sinus Lift Surgery. Am. J. Rhinol..

[43] Ting M., Rice J.G., Braid S.M., Lee C.Y.S., Suzuki J.B. (2017). Maxillary Sinus Augmentation for Dental Implant Rehabilitation of the Edentulous Ridge: A Comprehensive Overview of Systematic Reviews. Implant Dent..

[44] Miki M., Botticelli D., Silva E., Xavier S., Baba S. (2021). Incidence of Sinus Mucosa Perforations During Healing After Sinus Elevation Using Deproteinized Bovine Bone Mineral as Grafting Material: A Histologic Evaluation in a Rabbit Model. Int. J. Oral Maxillofac. Implant..

[45] Nakajima Y., Botticelli D., De Rossi E.F., Balan V.F., Godoy E.P., Silva E.R., Xavier S.P. (2023). Schneiderian Membrane Collateral Damage Caused by Collagenated and Non-Collagenated Xenografts: A Histological Study in Rabbits. Dent. J..

[46] Maniwa N., Xavier S.P., de Souza S.L.S., Silva E.R., Botticelli D., Morinaga K., Baba S. (2024). Sequential Bone Repair in Rabbit Sinus Lifts Using Bio-Oss and Hyaluronic Acid–Polynucleotide Gel (Regenfast). J. Funct. Biomater..

[47] Yamada R., Xavier S.P., Nakajima Y., Silva E.R., Botticelli D., Teranishi Y., Baba S. (2025). Impact of Collagenated and Non-Collagenated Deproteinized Bovine Bone Mineral on Schneiderian Membrane Integrity in Rabbits. Dent. J..

[48] Nakajima Y., Alccayhuaman K.A.A., Botticelli D., Lang N.P., De Rossi E.F., Xavier S.P. (2023). Mucosal adhesion phenomenon after maxillary sinus floor elevation: A preclinical study. Clin. Oral Implant. Res..

[49] Amari Y., Botticelli D., Alccayhuaman K., Hirota A., Silva E., Xavier S. (2020). The Influence on Healing of Bony Window Elevated Inward in the Sinus Cavity as Cortical Bone Graft: A Histomorphometric Study in Rabbit Model. Int. J. Oral Maxillofac. Implant..

[50] Favero R., Alccayhuaman K.A.A., Botticelli D., Xavier S.P., Balan V.F., Macchi V., De Caro R. (2021). Sinus Mucosa Thinning and Perforations after Sinus Lifting Performed with Different Xenografts: A Histological Analysis in Rabbits. Dent. J..

[51] Iida T., Neto E.C.M., Botticelli D., Alccayhuaman K.A.A., Lang N.P., Xavier S.P. (2017). Influence of a collagen membrane positioned subjacent the sinus mucosa following the elevation of the maxillary sinus. A histomorphometric study in rabbits. Clin. Oral Implant. Res..

[52] Figueiredo M., Henriques J., Martins G., Guerra F., Judas F., Figueiredo H. (2009). Physicochemical characterization of biomaterials commonly used in dentistry as bone substitutes—Comparison with human bone. J. Biomed. Mater. Res. Part B Appl. Biomater..

[53] Lee J.H., Yi G.S., Lee J.W., Kim D.J. (2017). Physicochemical characterization of porcine bone-derived grafting material and comparison with bovine xenografts for dental applications. J. Periodontal. Implant. Sci..

[54] Trajkovski B., Jaunich M., Müller W.-D., Beuer F., Zafiropoulos G.-G., Houshmand A. (2018). Hydrophilicity, Viscoelastic, and Physicochemical Properties Variations in Dental Bone Grafting Substitutes. Materials.

[55] Fujioka-Kobayashi M., Müller H.-D., Mueller A., Lussi A., Sculean A., Schmidlin P.R., Miron R.J. (2017). In vitro effects of hyaluronic acid on human periodontal ligament cells. BMC Oral Health.

[56] Pesce P., Menini M., Canullo L., Khijmatgar S., Modenese L., Gallifante G., Del Fabbro M. (2021). Radiographic and Histomorphometric Evaluation of Biomaterials Used for Lateral Sinus Augmentation: A Systematic Review on the Effect of Residual Bone Height and Vertical Graft Size on New Bone Formation and Graft Shrinkage. J. Clin. Med..

[57] Corbella S., Taschieri S., Weinstein R., Del Fabbro M. (2015). Histomorphometric outcomes after lateral sinus floor elevation procedure: A systematic review of the literature and meta-analysis. Clin. Oral Implant. Res..

[58] Taniguchi Z., Esposito M., Xavier S.P., Silva E.R., Botticelli D., Buti J., Baba S. (2024). On The Use of a Sticky Bone Substitute in the Presence of a Ruptured Schneider Membrane During Sinus Lift Procedures—An Experimental Within-Rabbit Study. Int. J. Oral Maxillofac. Implant..

[59] Lim H., Son Y., Hong J., Shin S., Jung U., Chung J. (2018). Sinus floor elevation in sites with a perforated schneiderian membrane: What is the effect of placing a collagen membrane in a rabbit model?. Clin. Oral Implant. Res..

[60] Ekşi C., Şeker B. (2025). Evaluating the relationship between periodontal bone loss in maxillary posterior teeth and Schneiderian membrane thickness. BMC Oral Health.

[61] Ellegaard B., Kølsen-Petersen J., Baelum V. (1997). Implant therapy involving maxillary sinus lift in periodontally compromised patients. Clin. Oral Implant. Res..

[62] Lundgren S., Andersson S., Gualini F., Sennerby L. (2004). Bone reformation with sinus membrane elevation: A new surgical technique for maxillary sinus floor augmentation. Clin. Implant. Dent. Relat. Res..

[63] Ellegaard B., Baelum V., Kølsen-Petersen J. (2006). Non-grafted sinus implants in periodontally compromised patients: A time-to-event analysis. Clin. Oral Implant. Res..

[64] Cricchio G., Sennerby L., Lundgren S. (2011). Sinus bone formation and implant survival after sinus membrane elevation and implant placement: A 1- to 6-year follow-up study. Clin. Oral Implant. Res..

[65] Lundgren S., Johansson A.S., Cricchio G., Lundgren S. (2019). Clinical outcome and factors determining new bone formation in lateral sinus membrane elevation with simultaneous implant placement without grafting material: A cross-sectional, 317 year follow-up study. Clin. Implant. Dent. Relat. Res..

[66] Carmagnola D., Pispero A., Pellegrini G., Sutera S., Henin D., Lodi G., Achilli A., Dellavia C. (2024). Maxillary sinus lift augmentation: A randomized clinical trial with histological data comparing deproteinized bovine bone grafting vs. graftless procedure with a 5–12-year follow-up. Clin. Implant. Dent. Relat. Res..

[67] Omori Y., Botticelli D., Ferri M., Delgado-Ruiz R., Balan V.F., Xavier S.P. (2021). Argon Bioactivation of Implants Installed Simultaneously to Maxillary Sinus Lifting without Graft. An Experimental Study in Rabbits. Dent. J..

